# Association between pulmonary function and balance, motor function, and trunk stability in stroke survivors

**DOI:** 10.3389/fmed.2026.1827402

**Published:** 2026-06-18

**Authors:** Shuai Guo, Anming Hu, Yumei Zhang

**Affiliations:** Department of Rehabilitation Medicine, Beijing Tiantan Hospital, Capital Medical University, Beijing, China

**Keywords:** balance, motor function, pulmonary function, stroke, trunk stability

## Abstract

**Background:**

Post-stroke rehabilitation is often delivered in a fragmented manner across disciplines, with respiratory function routinely overlooked. This oversight represents a significant gap in comprehensive stroke management, potentially limiting functional recovery, and undermining efforts to deliver truly integrated, patient-centered rehabilitation.

**Objective:**

This study aims to provide empirical evidence supporting the integration of pulmonary assessment into multidisciplinary stroke rehabilitation by examining, in stroke survivors, the associations between standard pulmonary function parameters and key functional domains, including objective measures of balance, lower-limb motor function, and trunk stability.

**Methods:**

In this observational study, 46 patients with stroke underwent comprehensive assessments, which included pulmonary function [percent predicted vital capacity (VC%), inspiratory capacity (IC%), forced vital capacity (FVC%), forced expiratory volume in 1 s (FEV1%), ratio of FEV1 to FVC (FEV1/FVC), peak expiratory flow (PEF%), maximum voluntary ventilation (MVV%)], the Berg Balance Scale (BBS), the Fugl-Meyer Assessment for Lower Extremity (FMA-LE), the Trunk Impairment Scale (TIS), and plantar pressure analysis [center of pressure (COP) path length, COP velocity, and 95% confidence ellipse area].

**Results:**

In this exploratory analysis, the BBS and FMA-LE scores showed positive correlations with VC%, FEV1%, and PEF% (*r* = 0.30–0.48, *p* < 0.05). TIS scores were positively associated with multiple pulmonary parameters (VC%, IC%, FVC%, FEV1%, FEV1/FVC, PEF%, MVV%; *r* = 0.31–0.54, *p* < 0.05), with the strongest exploratory correlation observed with PEF% (*r* = 0.54, *p* < 0.001). Furthermore, PEF% was negatively correlated with COP path length and velocity (*r*_*s*_ = −0.30 to −0.33, *p* < 0.05). The 95% confidence ellipse area was negatively correlated with IC% (*r*_*s*_ = −0.40, *p* < 0.01) and PEF% (*r*_*s*_ = −0.34, *p* < 0.05).

**Conclusion:**

Our results support the integration of simple, low-cost pulmonary screening, particularly PEF measurement, into stroke rehabilitation workflows. Such integration can act as a practical trigger for timely multidisciplinary collaboration among general practitioners, respiratory therapists, and rehabilitation specialists, thereby fostering a more cohesive, proactive approach to improving long-term functional trajectories in stroke survivors.

## 1 Introduction

Stroke is the leading cause of death and disability among adults, imposing a substantial societal and familial burden (1). Over two-thirds of survivors experience persistent, interrelated impairments, including balance dysfunction, lower-limb motor deficits, trunk instability, and compromised respiratory function, which collectively limit activities of daily living (ADLs) and rehabilitation outcomes (2, 3). Central nervous system (CNS) damage, particularly cerebral lesions, not only disrupts trunk muscle strength and coordination (3) but also exerts specific effects on respiratory control. Evidence suggests that supratentorial brain lesions can impair the bilateral neural drive to the diaphragm, leading to asymmetric diaphragmatic thinning and reduced excursion. This effect is particularly pronounced on the hemiparetic side during deep inspiration (4). Consequently, pulmonary parameters such as forced vital capacity (FVC), forced expiratory volume in 1 s (FEV1), and peak expiratory flow (PEF) are consistently lower post-stroke than in healthy controls (5), contributing to hypoxemia, impaired airway clearance, reduced exercise tolerance, and diminished rehabilitation intensity.

Critically, respiration and postural control are biomechanically linked: the diaphragm stabilizes the trunk via intra-abdominal pressure modulation, supporting anticipatory postural adjustments (4). In older adults and chronic disease populations, FVC and FEV1 correlate with balance performance, likely due to oxygen delivery limitations affecting muscle efficiency (6). COPD studies further show that poor pulmonary function predicts motor decline (6, 7). Notably, respiratory training in stroke patients improves not only lung function but also balance, motor function, and trunk control (8, 9), suggesting cross-domain benefits. However, the specific biomechanical mechanisms underpinning this association remain poorly elucidated. While interventions demonstrate efficacy, there is a paucity of objective evidence linking standard pulmonary metrics to quantifiable biomechanical parameters of postural stability, such as center of pressure (COP) displacement or trunk stability. Therefore, investigating the correlation between pulmonary function and objective biomechanical indicators (including plantar pressure distribution and trunk control) is essential to validate the physiological basis of this cross-domain interaction.

Yet, despite this evidence, pulmonary assessment is rarely integrated into routine stroke rehabilitation. Accordingly, we investigate associations between pulmonary function and key domains, including balance, motor function, and trunk stability in stroke survivors using objective plantar pressure analysis. By linking respiratory metrics to quantifiable functional indicators, we aim to support the integration of pulmonary screening into multidisciplinary rehabilitation pathways to optimize long-term outcomes.

## 2 Materials and methods

### 2.1 Study design and patients

This was a non-interventional, cross-sectional observational study conducted at the Department of Rehabilitation Medicine, Beijing Tiantan Hospital, Capital Medical University, between October 2021 and October 2023. The study was conducted and reported in accordance with the Strengthening the Reporting of Observational Studies in Epidemiology (STROBE) guidelines for cross-sectional studies. The completed STROBE checklist for this cross-sectional study is available as Supplementary Data Sheet 1. All participants underwent assessments of balance, motor function, trunk stability, pulmonary function, and plantar pressure distribution.

Inclusion criteria were as follows:

(1) First-ever clinical stroke episode;

(2) Confirmed diagnosis of stroke supported by head computed tomography (CT) or magnetic resonance imaging (MRI) findings;

(3) Age between 18 and 80 years;

(4) Time since stroke onset ≤ 3 months. This timeframe was chosen to focus on the subacute phase of recovery, during which functional improvements are typically most rapid. By concentrating on this dynamic period, we aimed to capture the associations between pulmonary function and motor/balance outcomes before potential plateauing of recovery, thereby reducing variability related to different stages of the recovery trajectory.

(5) Presence of unilateral lower-limb motor impairment;

(6) Lower-extremity motor recovery at Brunnstrom stages II–IV;

(7) Able to stand independently for ≥ 30 s.

Exclusion criteria included:

(1) Visual impairment, visual field deficits, or hemispatial neglect;

(2) Orthopedic deformities of the lower limbs or lumbar compression fractures;

(3) Posterior circulation stroke;

(4) Significant cognitive impairment [Montreal Cognitive Assessment (MoCA) score ≤ 22];

(5) Comorbid psychiatric disorders;

(6) Severe cardiovascular conditions (e.g., arrhythmias, myocardial infarction, or heart failure);

(7) Clinically significant cardiopulmonary diseases (e.g., chronic obstructive pulmonary disease, asthma, or respiratory failure);

(8) Presence of a tracheostomy or nasogastric tube.

This study was conducted in accordance with the principles of the Declaration of Helsinki and was approved by the Ethics Committee of Beijing Tiantan Hospital, Capital Medical University (Approval No. KY2020-069-01). Written informed consent was obtained from all participants or their legally authorized representatives prior to enrollment.

### 2.2 Measurement

All assessments were conducted during a single session lasting approximately 30–60 min, with sufficient rest periods provided between tests to avoid fatigue. At the time of evaluation, all participants were hospitalized in the Department of Rehabilitation Medicine at Beijing Tiantan Hospital and were undergoing routine post-stroke rehabilitation.

The following clinical and sociodemographic variables were systematically collected: age, sex, height, weight, body mass index (BMI), smoking status (yes vs. no), stroke type (ischemic vs. hemorrhagic), paretic side (left vs. right), time since stroke onset (days), and Brunnstrom lower-extremity stage.

All assessments were performed by a single trained therapist with more than 5 years of clinical experience, who underwent standardized training prior to the study. For each outcome measure, the mean of three repeated measurements was used unless otherwise specified.

#### 2.2.1 Pulmonary function assessment

Pulmonary function testing was conducted using a standardized spirometer (Nanjing Highermed, Model Emax58-0012, Nanjing, China). Prior to each testing session, the device was calibrated according to the manufacturer's instructions. Participants were seated in an upright position and wore a facemask connected to the spirometer. They were instructed to breathe normally first, and then to perform the required respiratory maneuvers (e.g., maximal inspiration followed by forceful exhalation) following verbal commands. Each test was repeated three times, and the best result was retained for analysis. Primary parameters assessed included vital capacity (VC), inspiratory capacity (IC), expiratory reserve volume (ERV), inspiratory reserve volume (IRV), forced vital capacity (FVC), forced expiratory volume in 1 s (FEV1), FEV1/FVC ratio, peak expiratory flow (PEF), and maximum voluntary ventilation (MVV).

To account for the well-established influence of age, sex, height, weight, and ethnicity on pulmonary function metrics, absolute values were converted to percentages of predicted normal values (% predicted) using established reference equations. The reference equations used were those proposed by the Global Lung Function Initiative (GLI-2012) for the Asian population (10). These equations provide sex-specific and age-specific reference values and are widely accepted for multi-ethnic populations.

#### 2.2.2 Balance assessment

The Berg Balance Scale (BBS), widely regarded as the criterion standard for clinical balance assessment, has demonstrated excellent psychometric properties in stroke populations, with intra-rater ICC values of 0.97–0.99 and inter-rater ICC values of 0.96–0.98 (11). The scale comprises 14 items assessing balance during functional tasks of increasing difficulty: sitting unsupported, standing unsupported, sitting to standing, transferring, standing with eyes closed, standing with feet together, reaching forward, retrieving an object from the floor, turning to look behind, turning 360°, placing an alternating foot on a stool, tandem standing, and standing on one leg. Each item is scored from 0 (unable to perform) to 4 (independent performance), with a maximum total score of 56. Higher scores indicate better balance.

#### 2.2.3 Motor function assessment

The lower extremity subscale of the Fugl-Meyer Assessment (FMA-LE) was used to evaluate motor impairment in the affected limb. This validated clinical tool quantifies motor deficits across seven domains: reflex activity, flexor synergistic movements, extensor synergistic movements, combination of synergies, movement out of synergy, presence of hyperreflexia, and coordination/speed. The scale consists of 17 items, with a maximum possible score of 34; lower scores indicate greater severity of motor impairment.

#### 2.2.4 Trunk stability assessment

The Trunk Impairment Scale (TIS) was administered. The scale comprises three subscales: Static Sitting Balance (Items 1–3), Dynamic Sitting Balance (Items 4–10), and Trunk Coordination (Items 11–17), with a maximum total score of 23. Higher scores indicate better trunk control. The TIS shows strong clinimetric properties, including high internal consistency (Cronbach's alpha = 0.65–0.89) and excellent inter-rater reliability (ICC = 0.99), supporting its use in both clinical practice and research (12).

#### 2.2.5 Plantar pressure assessment

The Zebris plantar pressure measurement system (Zebris FDM, version 1.12) was used to assess postural sway during quiet standing. Participants were instructed to remove their shoes and socks and to stand barefoot on the force platform with arms relaxed at their sides and gaze directed straight ahead. They were asked to remain as still as possible throughout the trial. After achieving a stable upright posture for 30 s, data acquisition was initiated and continued for 10 s. The procedure was repeated three times to ensure reliability.

The primary outcome parameters included: (1) center of pressure (COP) path length, (2) COP velocity, and (3) 95% confidence ellipse area. These metrics provide objective, quantitative measures of postural sway during quiet standing. A shorter COP path length reflects improved static balance control, while reduced COP velocity and a smaller 95% confidence ellipse area collectively indicate enhanced postural stability and diminished sway amplitude (13–15).

### 2.3 Statistical analysis

#### 2.3.1 Sample size calculation

A priori power analysis was performed using G^*^Power software (version 3.1). According to the correlation strength guidelines proposed by Schober et al., correlations of *r* ≥ 0.40 are considered moderate and clinically meaningful. To detect a moderate correlation coefficient of *r* = 0.40 with a significance level (α) of 0.05 and a statistical power (1–β) of 0.80, a minimum sample size of 47 participants was required. Our final sample of 46 participants was slightly below this estimate, achieving an actual power of approximately 79%. Therefore, this study should be considered exploratory; the findings provide preliminary evidence, and the observed moderate-to-strong correlations (*r* ≥ 0.40) should be interpreted with appropriate caution.

Data were analyzed using IBM SPSS Statistics version 23.0 (IBM Corp., Armonk, NY, USA). Categorical variables are reported as frequencies and percentages. Continuous variables are presented as mean ± standard deviation (SD) for normally distributed data and as median [interquartile range (IQR)] for non-normally distributed data. No imputation was performed for missing data; participants with incomplete assessments were excluded from the final analysis, as described in Section 3 (*n* = 15). Bivariate correlations were evaluated using Pearson's correlation coefficient (*r*) for normally distributed variables and Spearman's rank correlation coefficient (*r*_*s*_) for non-normally distributed variables. The correlation coefficients (*r* or *r*_*s*_) and corresponding *p-values* are reported to indicate the strength and significance of the associations. The strength of the correlation coefficients was interpreted based on the following guidelines: negligible (0.00–0.10), weak (0.10–0.39), moderate (0.40–0.69), strong (0.70–0.89), and very strong (0.90–1.00). Statistical significance was set at a two-tailed *p* < 0.05.

## 3 Results

**Figure 1:**
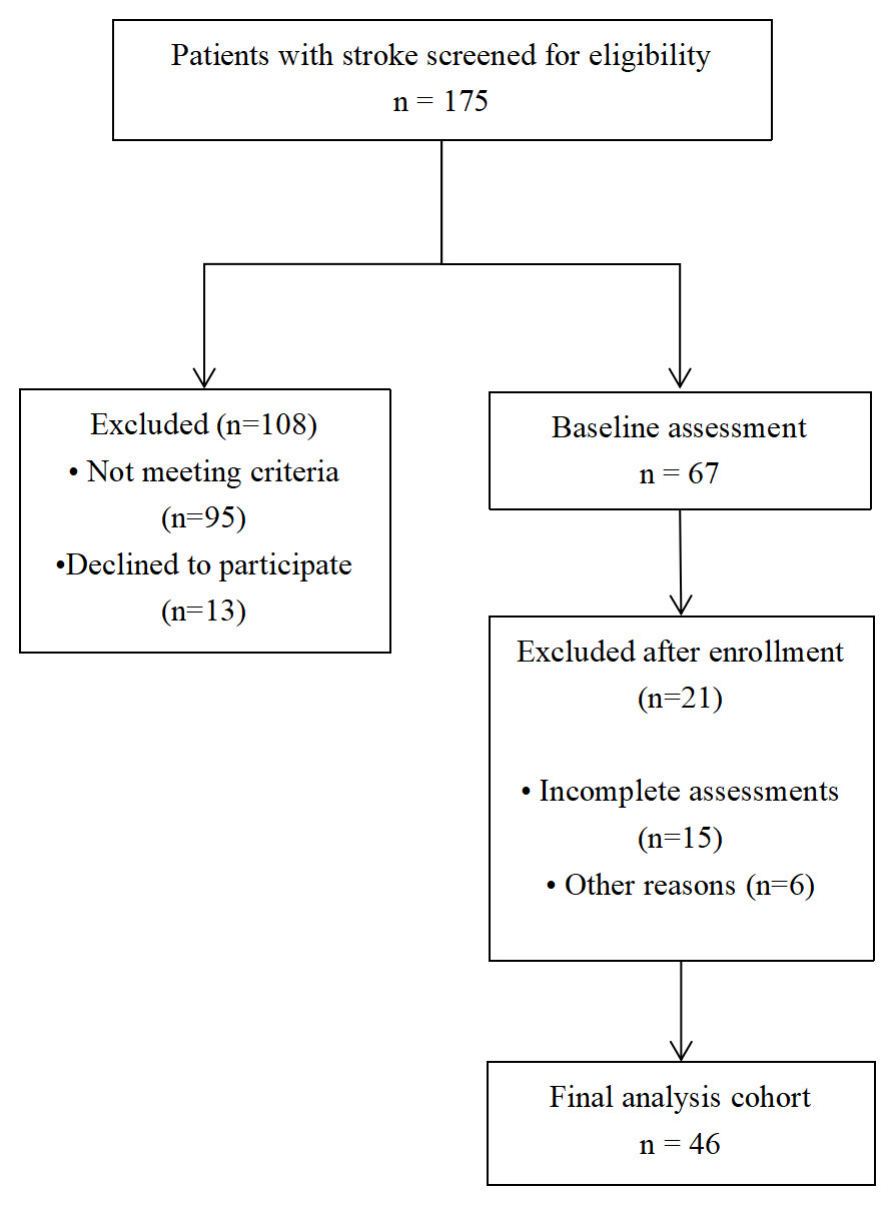
Flowchart of patient selection and study design. A total of 175 patients with stroke were initially screened. After applying exclusion criteria (e.g., severe cognitive impairment, inability to stand independently, history of respiratory disease), 46 patients were included in the final analysis. Participants underwent pulmonary function tests, balance assessment, lower limb motor function assessments, trunk impairment evaluations, and plantar pressure measurements.

A total of 175 stroke patients were screened from the Department of Rehabilitation Medicine at Beijing Tiantan Hospital, Capital Medical University, between October 2021 and October 2023. Following application of the predefined inclusion and exclusion criteria, 67 eligible participants were initially enrolled. Of these, 21 were subsequently excluded due to incomplete assessments (*n* = 15) or other reasons (*n* = 6), resulting in a final analytic cohort of 46 participants (Figure 1).

Demographic and clinical characteristics are shown in Table 1. The 46 stroke survivors (mean age 55.26 ± 10.95 years; 71.7% male) were predominantly ischemic (93.5%), with equal left/right lesion distribution and a median post-stroke duration of 16 days. As shown in Table 2, participants exhibited moderate balance impairment (BBS: 29.76 ± 12.26), mild-to-moderate motor deficits (FMA-LE: 20.02 ± 6.59), and reduced pulmonary function, particularly PEF% (50.96 ± 20.77%).

**Table 1:** Baseline characteristics of enrolled stroke patients (*n* = 46).

Characteristic	Value
Age (years), mean ± SD	55.26 ± 10.95
Sex, *n* (%)	
Male	33 (71.7)
Female	13 (28.3)
Height (m), mean ± SD	1.67 ± 0.07
Weight (kg), mean ± SD	74.35 ± 14.06
BMI (kg/m^2^), mean ± SD	26.37 ± 3.39
**Smoking status**, ***n*** **(%)**	
Yes	18 (39.1)
No	28 (60.9)
**Stroke type**, ***n*** **(%)**	
Ischemic	43 (93.5)
Hemorrhagic	3 (6.5)
**Paretic side**, ***n*** **(%)**	
Left	23 (50.0)
Right	23 (50.0)
**Brunnstrom lower extremity stage**, ***n*** **(%)**	
II	11(23.9)
III	27(58.7)
IV	8(17.4)
Post-stroke duration (days), median (IQR)	16.0 (12.0–38.0)

BMI, body mass index. All values are reported in SI units where applicable.

**Table 2:** Evaluation of functional assessments, plantar pressure metrics, and pulmonary function (*n* = 46).

Characteristic	Value
Functional assessments, mean ±SD	
BBS (0–56)	29.76 ± 12.26
FMA-LE (0–34)	20.02 ± 6.59
TIS (0–23)	11.48 ± 2.35
Plantar pressure metrics, median (IQR)	
COP path length (mm)	132.5 (89.75–160.0)
COP velocity (mm/s)	13.5 (9.0–16.0)
95% confidence ellipse area (mm^2^)	258.5 (170.5–472.0)
**Pulmonary function, mean** ±**SD**	
VC%	72.79 ± 12.98
IC%	90.95 ± 18.66
ERV%	24.24 ± 20.51
IRV (L)	1.73 ± 0.54
FVC%	72.53 ± 13.06
FEV1%	67.34 ± 17.13
FEV1/FVC%	70.30 ± 14.96
PEF%	50.96 ± 20.77
MVV%	75.05 ± 27.40

BBS, Berg Balance Scale; FMA-LE, Fugl-Meyer Assessment for Lower Extremity; TIS, Trunk Impairment Scale; COP, center of pressure; VC, vital capacity; IC, inspiratory capacity; ERV, expiratory reserve volume; IRV, inspiratory reserve volume; FVC, forced vital capacity; FEV1, forced expiratory volume in 1 s; FEV1/FVC, ratio of FEV1 to FVC; PEF, peak expiratory flow; MVV, maximum voluntary ventilation. % values indicate percent predicted. IRV is presented in absolute values (liters) as there are no universally accepted reference equations for percent predicted IRV in this population. All values are reported in SI units where applicable.

### 3.1 Correlation analysis between pulmonary function and BBS, FMA-LE, TIS, and plantar pressure

**Figure 2:**
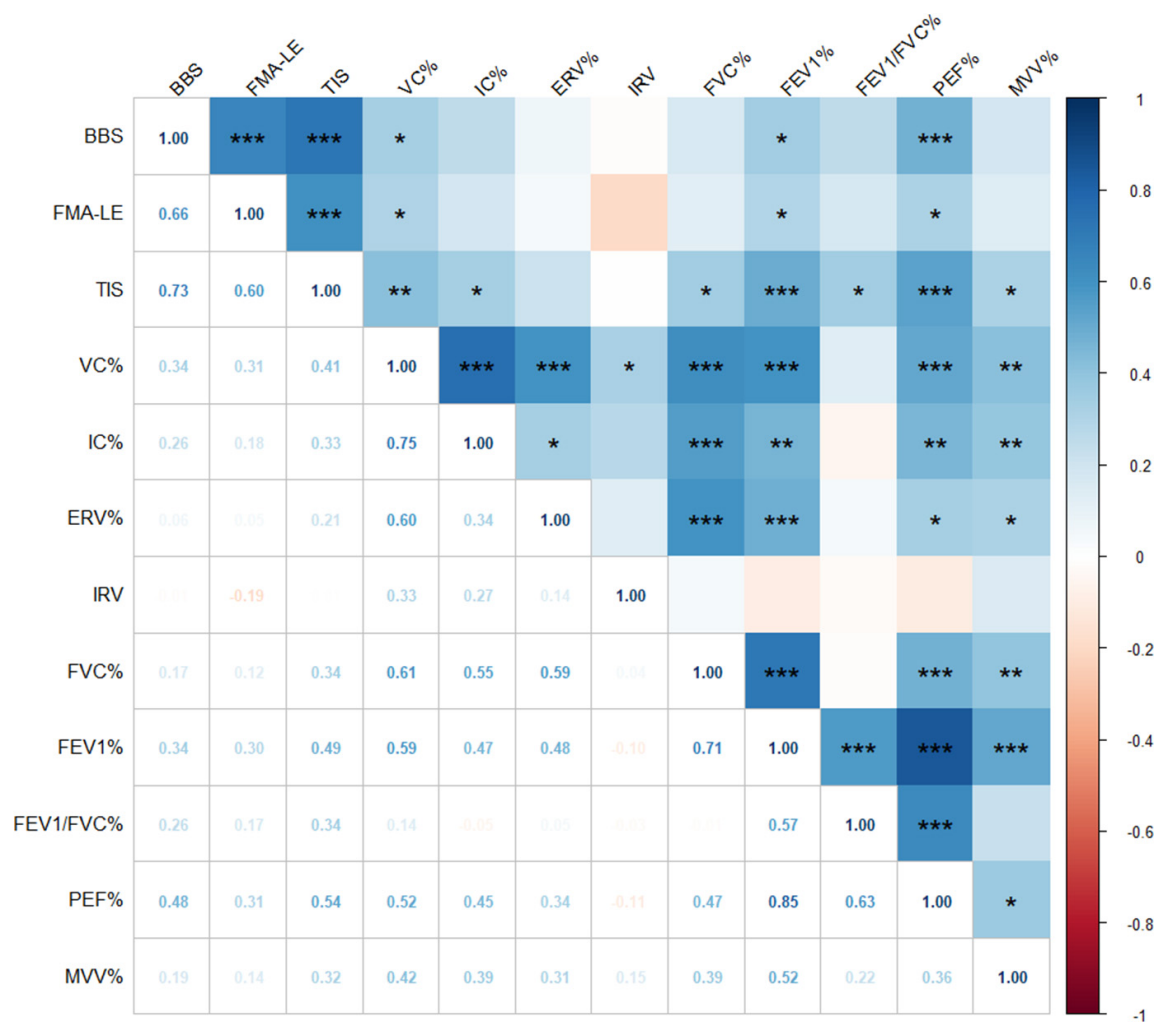
Heatmap of Pearson correlation coefficients between pulmonary function parameters and functional assessment scores (BBS, FMA-LE, TIS) in 46 stroke patients. The color gradient represents the Pearson correlation coefficient (*r*) with values ranging from −1 (strong negative correlation) to 1 (strong positive correlation), correlation strength is interpreted as follows: negligible (0.00–0.10), weak (0.10–0.39), moderate (0.40–0.69), strong (0.70–0.89), and very strong (0.90–1.00). Statistical significance is indicated by asterisks: *p < 0.05, **p < 0.01, ***p < 0.001. BBS, Berg Balance Scale; FMA-LE, Fugl-Meyer Assessment for Lower Extremity; TIS, Trunk Impairment Scale; VC, vital capacity; IC, inspiratory capacity; ERV, expiratory reserve volume; IRV, inspiratory reserve volume; FVC, forced vital capacity; FEV1, forced expiratory volume in 1 s; FEV1/FVC: ratio of FEV1 to FVC; PEF: peak expiratory flow; MVV, maximum voluntary ventilation.

**Figure 3:**
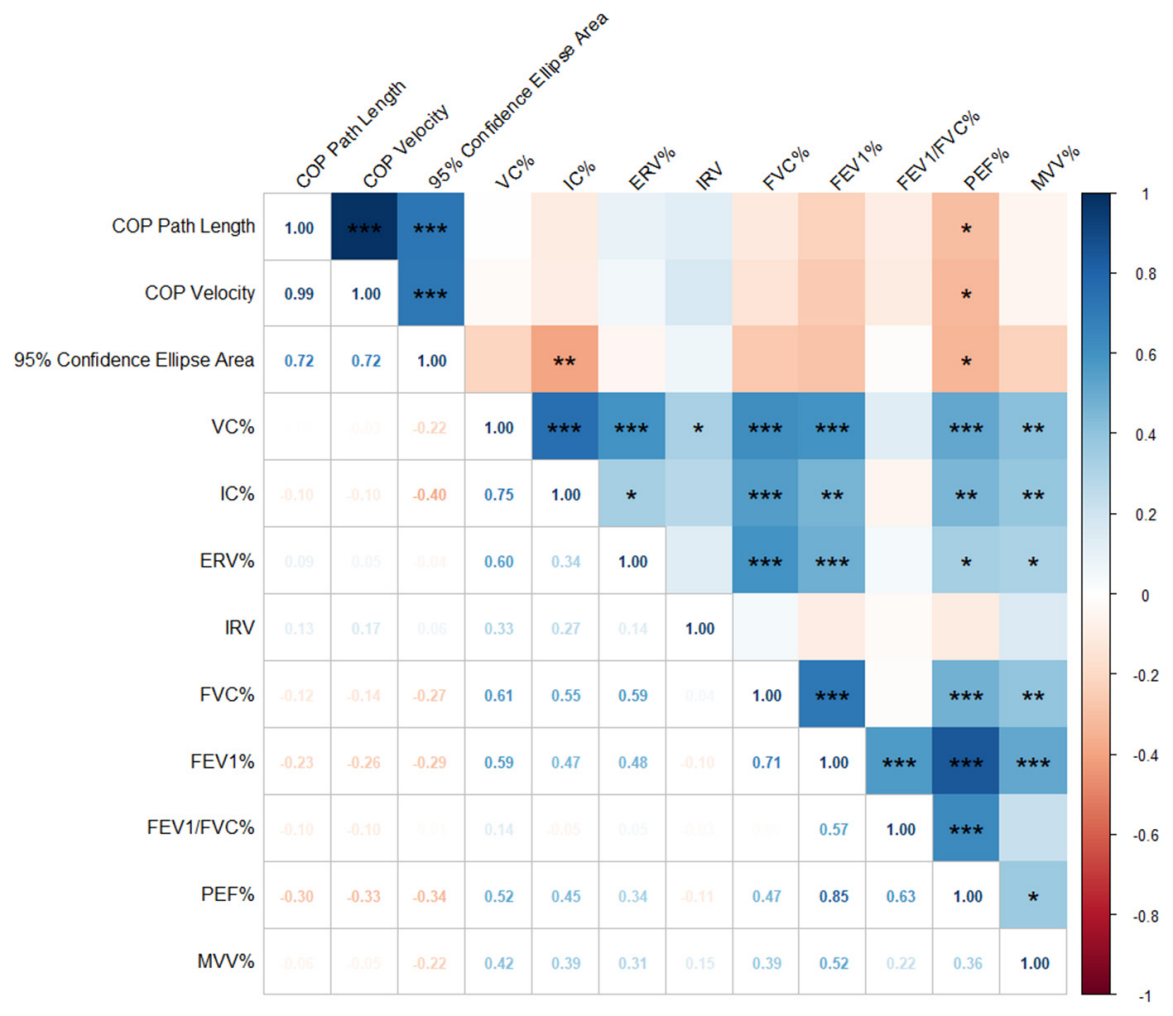
Heatmap of Spearman correlation coefficients between pulmonary function parameters and plantar pressure (COP path length, COP velocity, 95% confidence ellipse area) in 46 stroke patients. The color gradient represents the Spearman correlation coefficient (*r*_*s*_) with values ranging from −1 (strong negative correlation) to 1 (strong positive correlation), correlation strength is interpreted as follows: negligible (0.00–0.10), weak (0.10–0.39), moderate (0.40–0.69), strong (0.70–0.89), and very strong (0.90–1.00). Statistical significance is indicated by asterisks: *p < 0.05, **p < 0.01, ***p < 0.001. COP, center of pressure; VC, vital capacity; IC, inspiratory capacity; ERV, expiratory reserve volume; IRV, inspiratory reserve volume; FVC, forced vital capacity; FEV1, forced expiratory volume in 1 s; FEV1/FVC, ratio of FEV1 to FVC; PEF, peak expiratory flow; MVV, maximum voluntary ventilation.

The results of the correlation analyses are presented in Figures 2 and 3. The BBS score showed weak-to-moderate positive correlations with VC% (*r* = 0.34, *p* < 0.05) and FEV1% (*r* = 0.34, *p* < 0.05), and a moderate positive correlation with PEF% (*r* = 0.48, *p* < 0.001). The FMA-LE score was correlated with VC% (*r* = 0.31), FEV1% (*r* = 0.30), and PEF% (*r* = 0.31), all of weak strength (*p* < 0.05 for each). The TIS total score showed weak-to-moderate positive correlations with VC% (*r* = 0.41), IC% (*r* = 0.33), FVC% (*r* = 0.34), FEV1% (*r* = 0.49), FEV1/FVC (*r* = 0.34), and MVV% (*r* = 0.32), and a moderate positive correlation with PEF% (*r* = 0.54) (all *p* < 0.05, except FEV1% and PEF% *p* < 0.001).

Conversely, Spearman's analysis revealed weak negative correlations between PEF% and COP path length (*r*_*s*_ = −0.30, *p* < 0.05) and COP velocity (*r*_*s*_ = −0.33, *p* < 0.05). The 95% confidence ellipse area showed weak negative correlations with PEF% (*r*_*s*_ = −0.34, *p* < 0.05) and moderate negative correlation with IC% (*r*_*s*_ = −0.40, *p* < 0.01).

Given the exploratory nature of this study and the lack of correction for multiple comparisons, these weak correlations and borderline *p-values* should be interpreted with caution and considered hypothesis-generating rather than confirmatory.

## 4 Discussion

A growing body of evidence highlights the intricate interplay between pulmonary function and key functional outcomes, including balance, motor function of the lower limb, and trunk stability in individuals post-stroke. Notably, respiratory muscles, particularly the diaphragm and intercostal muscles, also serve as integral components of the postural control system. Impaired activation or dysfunction of these muscles can diminish core stability, thereby adversely affecting trunk control and balance (16). A deeper understanding of these multisystem interactions is essential for developing comprehensive, integrated rehabilitation approaches that simultaneously target respiratory and neuromuscular impairments. Such strategies hold the potential to optimize functional recovery and meaningfully improve patients' quality of life.

Based on data from our cohort of 46 stroke patients and supported by established physiological principles, the significant positive correlations observed between pulmonary function and multiple functional outcomes can be mechanistically interpreted through the lens of core stability biomechanics. These findings not only corroborate prior literature but also provide a unifying framework linking respiratory physiology with neuromuscular control. The TIS demonstrated significant positive correlations with multiple percent-predicted pulmonary parameters, including VC%, IC%, FVC%, FEV1%, FEV1/FVC ratio, PEF%, and MVV% (*r* = 0.31–0.54, *p* < 0.05). This association is physiologically grounded in the dual role of core musculature, particularly the diaphragm and transversus abdominis, as both respiratory effectors and postural stabilizers (17–22). Their coordinated co-contraction generates intra-abdominal pressure (IAP), which serves as the biomechanical cornerstone of trunk stability (23, 24). Consequently, impaired pulmonary function following stroke often reflects diminished capacity to generate adequate IAP, directly undermining trunk control and manifesting as lower TIS scores. Our results align with those of Li et al. (25), Boz et al. (26), and Ozhan et al. (27), who similarly reported associations between TIS performance and indices of respiratory muscle strength or pulmonary function (e.g., PEF, FVC), collectively reinforcing the intimate physiological coupling between respiration and trunk stability. We further observed significant positive correlations between VC%, FEV1%, and PEF% and both BBS and FMA-LE scores (*r* = 0.30-−0.48, *p* < 0.05). The underlying mechanism involves neuromuscular synergy between the respiratory and postural systems: diaphragmatic contraction not only drives ventilation but also contributes to IAP generation, which supports dynamic trunk control during movement through central nervous system regulation and coordination with spinal stabilizers (28). During rapid postural adjustments, sufficient IAP is essential for the effective recruitment of superficial abdominal muscles (e.g., rectus abdominis, obliques) to maintain equilibrium. Direct evidence for this link comes from Liu et al. (29), who demonstrated that diaphragm function on the hemiplegic side correlates significantly with both limb motor recovery and balance performance, underscoring the diaphragm's active role in postural control beyond respiration. Moreover, the interventional study by Oh et al. (30) provides causal support: targeted respiratory muscle training enhanced respiratory strength and IAP, leading to measurable improvements in trunk stability and balance. Together, these findings offer a coherent explanation for the positive correlation between FEV1% and BBS observed in our cohort: namely, that pulmonary function and postural stability are functionally integrated through the shared pathway of IAP-mediated core stabilization.

A key methodological aspect of this study is the integration of objective biomechanical measures, specifically COP parameters, to quantify static standing balance, complementing traditional clinical rating scales. We found weak, statistically significant negative correlations between higher PEF% and reduced postural sway, as indicated by shorter COP path length (*r*_*s*_ = −0.30, *p* < 0.05), slower COP velocity (*r*_*s*_ = −0.33, *p* < 0.05), and a smaller 95% confidence ellipse area (*r*_*s*_ = −0.34, *p* < 0.05). Interpreted in the context of multiple comparisons, these findings should be viewed as exploratory. To our knowledge, this is the first study to report an inverse relationship between pulmonary function and objective measures of static postural sway in stroke survivors, although the strength of these associations was modest. Furthermore, we observed a moderate negative correlation between the 95% confidence ellipse area and IC% (*r*_*s*_ = −0.40, *p* < 0.01). This suggests that greater inspiratory capacity may be linked to enhanced postural stability. The finding aligns with the biomechanical model proposed by Hodges et al. (31), and while preliminary, it provides empirical data supporting the interdependence of respiratory mechanics and postural control in this population.

Notably, certain pulmonary function parameters, including ERV%, IRV, and MVV%, did not show significant associations with the functional assessments evaluated in this study. Several factors may account for this observation. First, these measures may be less sensitive than FEV1% or PEF% to the subtle respiratory–postural impairments present in the early-to-moderate stages of stroke recovery. Second, the modest sample size (*n* = 46) may have limited statistical power to detect weaker, yet potentially meaningful, correlations. Third, the physiological constructs captured by ERV%, IRV, and MVV%, such as ventilatory reserve or dynamic breathing capacity, may be less directly coupled to the specific functional domains assessed here (i.e., static balance, trunk control, and lower limb motor function) compared to indices reflecting airway flow dynamics (e.g., PEF%) or overall ventilatory efficiency (e.g., FEV1%). Future studies with larger cohorts and more granular respiratory profiling may help clarify the role of these parameters in post-stroke functional outcomes.

The present findings carry several practical implications for primary care–based stroke rehabilitation. First, given the significant correlations observed between PEF% and both trunk stability (TIS) and postural sway (COP parameters), handheld peak flow measurement emerges as a potential low-cost screening tool worthy of further investigation. Notably, our data showed that a substantial proportion of patients in this cohort exhibited PEF% values below 50% of predicted. This observation is hypothesis-generating and suggests that such low PEF% values might be associated with a higher likelihood of postural instability having an underlying respiratory component, thereby prompting earlier multidisciplinary input. However, the specific cutoff of 50% should be interpreted with caution and requires validation in future studies before it can be recommended for clinical use.

Several limitations of this study should be acknowledged. First, the cross-sectional design precludes the establishment of causal relationships between pulmonary function and balance, motor function, or trunk stability. While significant correlations were observed, it remains unclear whether impaired respiratory mechanics directly contribute to balance deficits or if both are parallel consequences of severe stroke impairment. Longitudinal studies or randomized controlled trials are needed to clarify the directionality of these associations and to determine whether respiratory muscle training can effectively improve balance outcomes. Second, although our sample size was sufficient to detect moderate-to-strong correlations (*r* ≥ 0.40), it may have had limited statistical power to detect weaker associations (e.g., *r* < 0.30). Consequently, the absence of significant correlations for certain pulmonary parameters (such as ERV% and IRV) should be interpreted with caution, as these may reflect type II error rather than a true absence of association. Third, we did not apply corrections for multiple comparisons (e.g., Bonferroni correction) in our correlation analyses; while this approach reduces the risk of Type II errors in this exploratory study, it may increase the likelihood of Type I errors (false positives). Fourth, the inclusion of participants across Brunnstrom lower-extremity stages II–IV may have introduced heterogeneity in motor recovery status, as stage II and stage IV represent distinct phases of recovery. However, the requirement that all participants stand independently for at least 30 seconds partly explains the relatively higher functional levels observed in this sample, as it selected higher-functioning individuals within each stage. Fifth, due to the modest sample size (*n* = 46) and the exploratory nature of this study, we did not perform multivariate regression analyses to adjust for potential confounding variables such as age, sex, or smoking status. Such analyses would have been underpowered and could lead to unreliable estimates or overfitting. Therefore, the observed associations should be interpreted as preliminary, and future larger-scale studies with appropriate covariate adjustments are needed to confirm these findings. Finally, our pulmonary function assessment relied on standard spirometry without direct measurement of respiratory muscle strength (e.g., maximal inspiratory/expiratory pressure), which might provide more specific insights into the mechanism linking respiration and trunk stability. Future studies with larger, multicenter cohorts and comprehensive respiratory muscle assessments are warranted to validate these preliminary findings.

## 5 Conclusion

In conclusion, within the limitations of a cross-sectional design and the statistical considerations regarding multiple comparisons, this study offers preliminary evidence suggesting that pulmonary function (particularly PEF%) may be associated with trunk stability and balance in stroke patients. These relationships are physiologically plausible given the dual role of respiratory muscles. Our findings tentatively support the integration of simple, low-cost pulmonary screening (e.g., handheld peak flow meter) into routine stroke rehabilitation as a potential trigger for multidisciplinary collaboration. Future prospective studies with larger samples and appropriate statistical corrections are needed to confirm these associations.

## Data Availability

The original contributions presented in the study are included in the article/Supplementary material, further inquiries can be directed to the corresponding author.
