# Supplementary material for: Association between pulmonary function and balance, motor function, and trunk stability in stroke survivors
Source: Front Med (Lausanne). 2026 Jun 18;13:1827402. doi: 10.3389/fmed.2026.1827402 (PMC13322942; doi:10.3389/fmed.2026.1827402)
Supplement: Supplementary file 1 [file Data_Sheet_1.pdf]

STROBE Statement—Checklist of items that should be included in reports of cross-sectional studies

Section/Topic	Item No	Recommendation	Reported on Page/Line
**Title and abstract**	1	(a) Indicate the study's design with a commonly used term in the title or the abstract	Page 1, Title; Page 1-2, Abstract
		(b) Provide in the abstract an informative and balanced summary of what was done and what was found	Page 1-2, Abstract
**Introduction**			
Background/rationale	2	Explain the scientific background and rationale for the investigation being reported	Page 3-4, Introduction
Objectives	3	State specific objectives, including any prespecified hypotheses	Page 4, Introduction, final paragraph
**Methods**			
Study design	4	Present key elements of study design early in the paper	Page 4, Section 2.1 ("cross-sectional observational study")
Setting	5	Describe the setting, locations, and relevant dates, including periods of recruitment	Page 4, Section 2.1 (Beijing Tiantan Hospital, October 2021–October 2023)
Participants	6	(a) Give the eligibility criteria, and the sources and methods of selection of participants	Page 4-5, Section 2.1 (inclusion and exclusion criteria)
Variables	7	Clearly define all outcomes, exposures, predictors, potential confounders, and effect modifiers	Page 5-8, Section 2.2 (pulmonary function, BBS, FMA-LE, TIS, COP parameters)
Data sources/measurement	8*	For each variable of interest, give sources of data and details of methods of assessment (measurement)	Page 5-8, Section 2.2
Bias	9	Describe any efforts to address potential sources of bias	Page 5, Section 2.2 (standardized training, repeated measurements, single assessor)
Study size	10	Explain how the study size was arrived at	Page 8, Section 2.3 (sample size calculation using G*Power)
Quantitative variables	11	Explain how quantitative variables were handled in the analyses	Page 8, Section 2.3 (normality assessment, mean±SD or median IQR)

Statistical methods	12	(a) Describe all statistical methods, including those used to control for confounding	Page 8, Section 2.3 (Pearson's r, Spearman's r_s)
		(b) Describe any methods used to examine subgroups and interactions	Not applicable
		(c) Explain how missing data were addressed	Page 8, Section 2.3 (participants with incomplete assessments excluded, n = 15)
		(d) If applicable, describe analytical methods taking account of sampling strategy	Not applicable
		(e) Describe any sensitivity analyses	Not performed (exploratory study)
**Results**			
Participants	13*	(a) Report numbers of individuals at each stage of study	Page 9, Results; Figure 1
		(b) Give reasons for non-participation at each stage	Page 9, Results; Figure 1
		(c) Consider use of a flow diagram	Figure 1
Descriptive data	14*	(a) Give characteristics of study participants and information on exposures and potential confounders	Table 1; Page 9, Results
		(b) Indicate number of participants with missing data for each variable of interest	Page 9, Results (n = 15 excluded due to incomplete assessments)
Outcome data	15*	Report numbers of outcome events or summary measures	Table 2
Main results	16	(a) Give unadjusted estimates and their precision (e.g., 95% CI)	95% CIs are not reported as this is an exploratory study without adjustment for multiple comparisons. Correlation coefficients (r or r_s) and p-values are reported in Figures 2-3 and in the Results text (Page 9-10).
		(b) Report category boundaries when continuous variables were categorized	Not applicable
		(c) If relevant, consider translating estimates of relative risk into absolute risk	Not applicable
Other analyses	17	Report other analyses done (subgroups, interactions, sensitivity analyses)	Not performed
**Discussion**			

Key results	18	Summarise key results with reference to study objectives	Page 12, Discussion, first paragraph
Limitations	19	Discuss limitations of the study, taking into account sources of potential bias or imprecision	Page 16-17, Discussion, limitations paragraph
Interpretation	20	Give a cautious overall interpretation of results considering objectives, limitations, and other relevant evidence	Page 14-17, Discussion
Generalisability	21	Discuss the generalisability (external validity) of the study results	Page 16-17, Discussion, limitations and future directions
**Other information**			
Funding	22	Give the source of funding and the role of the funders	Page 19, Funding section

STROBE Statement Declaration

This cross-sectional study was conducted and reported in accordance with the Strengthening the Reporting of Observational Studies in Epidemiology (STROBE) guidelines. The completed STROBE checklist is provided as supplementary material.
